# PRC1 chromatin factors strengthen the consistency of neuronal cell fate specification and maintenance in *C. elegans*

**DOI:** 10.1371/journal.pgen.1010209

**Published:** 2022-05-23

**Authors:** Guillaume Bordet, Carole Couillault, Fabien Soulavie, Konstantina Filippopoulou, Vincent Bertrand

**Affiliations:** Aix Marseille Univ, CNRS, IBDM, Turing Center for Living Systems, Marseille, France; University of Chicago Medical Center: The University of Chicago Medicine, UNITED STATES

## Abstract

In the nervous system, the specific identity of a neuron is established and maintained by terminal selector transcription factors that directly activate large batteries of terminal differentiation genes and positively regulate their own expression via feedback loops. However, how this is achieved in a reliable manner despite noise in gene expression, genetic variability or environmental perturbations remains poorly understood. We addressed this question using the AIY cholinergic interneurons of *C*. *elegans*, whose specification and differentiation network is well characterized. Via a genetic screen, we found that a loss of function of PRC1 chromatin factors induces a stochastic loss of AIY differentiated state in a small proportion of the population. PRC1 factors act directly in the AIY neuron and independently of PRC2 factors. By quantifying mRNA and protein levels of terminal selector transcription factors in single neurons, using smFISH and CRISPR tagging, we observed that, in PRC1 mutants, terminal selector expression is still initiated during embryonic development but the level is reduced, and expression is subsequently lost in a stochastic manner during maintenance phase in part of the population. We also observed variability in the level of expression of terminal selectors in wild type animals and, using correlation analysis, established that this noise comes from both intrinsic and extrinsic sources. Finally, we found that PRC1 factors increase the resistance of AIY neuron fate to environmental stress, and also secure the terminal differentiation of other neuron types. We propose that PRC1 factors contribute to the consistency of neuronal cell fate specification and maintenance by protecting neurons against noise and perturbations in their differentiation program.

## Author summary

During development, a high diversity of neuronal cell types is produced and they subsequently maintain their identity throughout the life of the animal. How this is achieved in a manner that is both accurate and robust to perturbations remains poorly understood. Terminal selector transcription factors play a key role in the specification and maintenance of neuron type identities. In this study, we observed that their expression is noisy. We also found that PRC1 chromatin factors contribute to the consistency of neuronal cell fate specification and maintenance. They help to set the correct level of terminal selector expression during the specification phase, and they secure the maintenance of terminal selector expression at later stages. PRC1 factors also increase the resistance of neuronal identity to environmental stress.

## Introduction

In animals, the nervous system is composed of a high diversity of neuronal cell types. The specific function and identity of a neuron are determined by the precise set of terminal differentiation genes (or effector genes) expressed by the neuron, such as neurotransmitter receptors or enzymes involved in neurotransmitter synthesis. Studies in vertebrates and invertebrates have shown that terminal differentiation genes are often coregulated by a common set of transcription factors, termed terminal selectors [1–3]. The expression of these terminal selectors is initiated during development in specific neuronal types by transient developmental inputs such as intercellular signaling or lineage inherited factors. They often subsequently maintain their own expression throughout the life of the neuron via positive feedback loops. Terminal selector transcription factors therefore play a key role in the specification of neuron type identities during development as well as in the maintenance of neuronal fates throughout the life of the animal.

For the nervous system to function properly it is key that the correct complement of neuronal cell types is generated during development and that they accurately maintain their identity afterwards. However, in various systems, quantitative analysis at single cell resolution have shown that the process of gene expression is noisy [4–6]. For instance, stochastic variability in the molecular interactions at the basis of transcription at a gene locus induces random fluctuations in the number of mRNA molecules produced. This leads to a variability of protein levels between otherwise identical cells in a homogenous environment, a phenomenon termed gene expression noise. These observations raise an intriguing question: how can the identity of a neuron be specified and maintained in a reliable manner despite variability in gene expression? In addition, neuronal fate has to be robust against internal (genetic) or external (environmental) perturbations.

*C*. *elegans* is a good system to study noise and precision during nervous system development. *C*. *elegans* has a fixed number of neurons (302 in the adult hermaphrodite) that are generated via an invariant cell lineage, allowing the monitoring of neuronal development with a high level of precision [7–10]. It is possible to work with isogenic populations and gene expression levels can be quantified at single cell resolution. The number of mRNA molecules of a specific gene in individual cells of *C*. *elegans* can be quantified with single molecule FISH (smFISH) [11]. In addition, CRISPR genome engineering at the endogenous locus allows tagging of endogenous proteins with GFP or RFP [12]. As *C*. *elegans* is transparent, the endogenous protein levels can then be quantified in single cells of individual animals by *in vivo* imaging.

**Fig 1:**
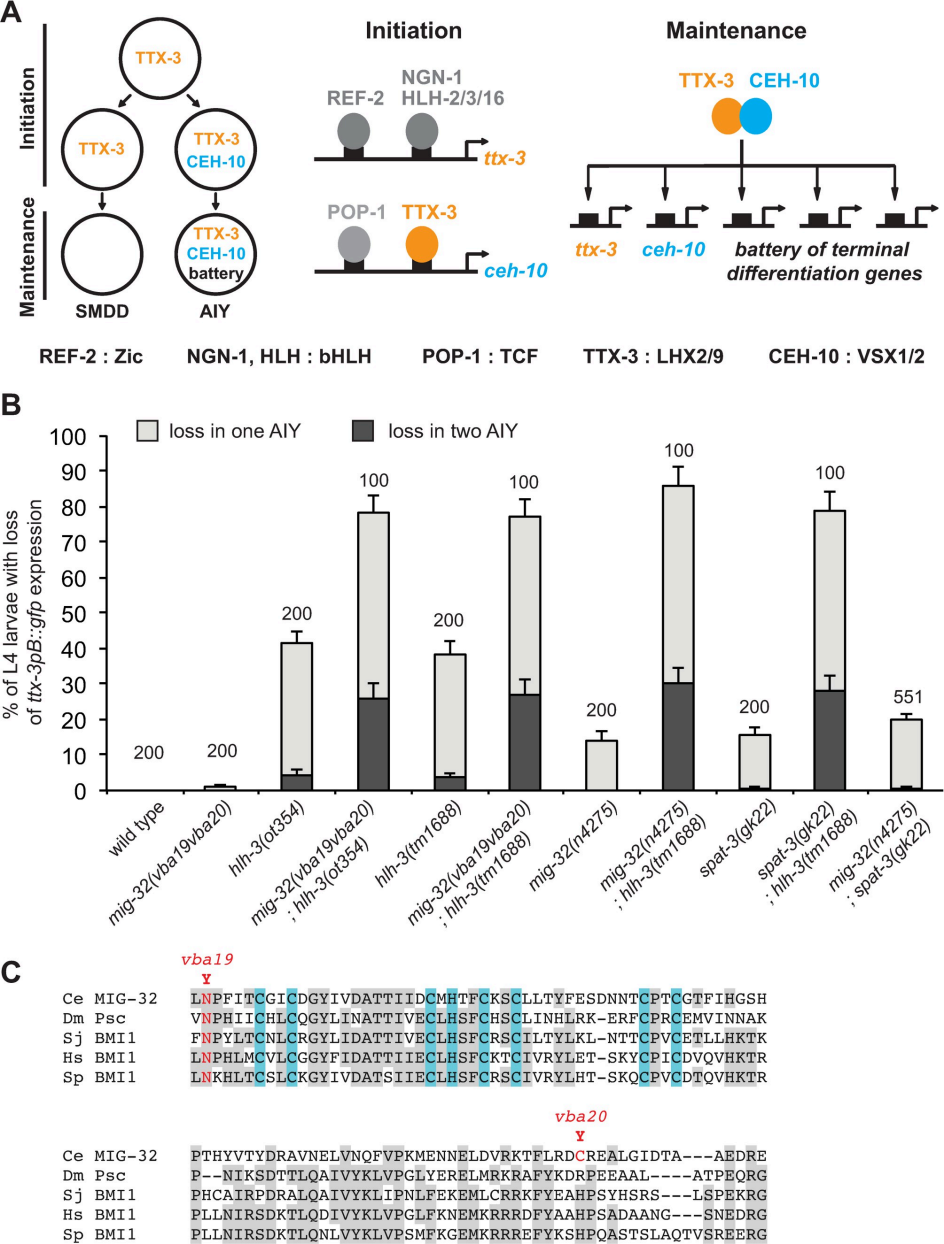
Isolation of a mutant in the PRC1 factor MIG-32 that affects the fate of the AIY neuron. (A) Gene regulatory network that initiates and maintains the type-specific identity of the AIY neuron. (B) Percentage of L4 larvae that display a loss of *ttx-3pB*::*gfp* (*otIs173*) expression in one (grey bar) or two (black bar) AIY neurons (error bars show standard error of proportion, numbers above the bars show number of animals analyzed). *mig-32; spat-3* double mutants are not statistically different from *mig-32* or *spat-3* single mutants (Fisher’s exact test, p>0.05). (C) Alignment of the RING finger and downstream region of MIG-32 with orthologs of the arthropod *Drosophila melanogaster* (Dm), the platyhelminth *Schistosoma japonicum* (Sj), the vertebrate *Homo sapiens* (Hs) and the echinoderm *Strongylocentrotus purpuratus* (Sp). The amino acids of the RING finger that bind Zn^2+^ are highlighted in blue. The two mutations present in *mig-32(vba19vba20)* are highlighted in red.

We focused on the pair of cholinergic interneurons AIY as their specification and differentiation network has been well characterized. The terminal differentiation of the AIY neuron is regulated by a complex of two terminal selectors, the homeodomain transcription factors TTX-3 (a LHX2/9 ortholog) and CEH-10 (a VSX1/2 ortholog) [13,14] (Fig 1A). During embryonic development, the expression of *ttx-3* is directly initiated in the AIY mother cell by a Zic transcription factor (REF-2) and several neural bHLH factors (HLH-2, HLH-3, HLH-16 and NGN-1) [15–17]. Following asymmetric division of the AIY mother cell, TTX-3 cooperates with an active Wnt pathway and its effector POP-1/TCF to directly activate the expression of *ceh-10* in the posterior daughter cell, the AIY neuron [15,18]. In the anterior daughter cell, the SMDD motor neuron, the Wnt pathway is not active and *ceh-10* is not expressed. Finally, in the AIY neuron, TTX-3 and CEH-10 directly activate and maintain the expression of a large battery of terminal differentiation genes such as choline acetyltransferase, acetylcholine vesicular transporter and various neurotransmitter receptors [13]. They also maintain their own expression via a positive autoregulatory loop. TTX-3 and CEH-10 therefore play a key role in the specification of the AIY neuron identity during embryogenesis and in its maintenance throughout the life of the animal.

In this paper, we identified, via a genetic screen, a role for Polycomb Repressive Complex 1 (PRC1) in the consistency of neuronal cell fate specification and maintenance. PRC1 is a chromatin-modifying complex that ubiquitylates histone H2A on lysine 119 [19,20]. While PRC1 factors have been historically implicated in gene repression, more recent data suggest that they can also play a role in gene activation [19–21]. The PRC1 complex often works with another Polycomb complex, PRC2, however cases where PRC1 works independently of PRC2 have also been observed [19–21]. The PRC1 complex is composed of a core of two proteins: RING1, the catalytic subunit, and PCGF (polycomb group ring finger), its cofactor. *C*. *elegans* has one RING1 protein (SPAT-3) and one PCGF (MIG-32) [22]. Only two studies have analyzed so far the role of PRC1 in *C*. *elegans* [22,23]. They identified a role in neuronal cell migration and axon guidance, however whether PRC1 factors also affect neuronal cell fate specification and maintenance is currently unknown.

In this paper, we show that PRC1 factors affect the differentiation program of the AIY neuron. We quantified, in single AIY neurons, the mRNA levels of the terminal selectors *ttx-3* and *ceh-10* using smFISH, as well as their protein levels using CRISPR tagging with fluorescent proteins. We observed that PRC1 factors are required to set the accurate level of terminal selector expression during neuronal specification in the embryo. Later, PRC1 factors are needed to ensure a 100% efficient maintenance of terminal selector expression in larvae and adult animals. We also show that PRC1 factors protect the AIY differentiation program against environmental stress, and contribute to the consistency of neuronal differentiation of other neuron types.

## Results

### Identification of PRC1 chromatin factors that affect *ttx-3* expression in the AIY neuron

**Fig 2:**
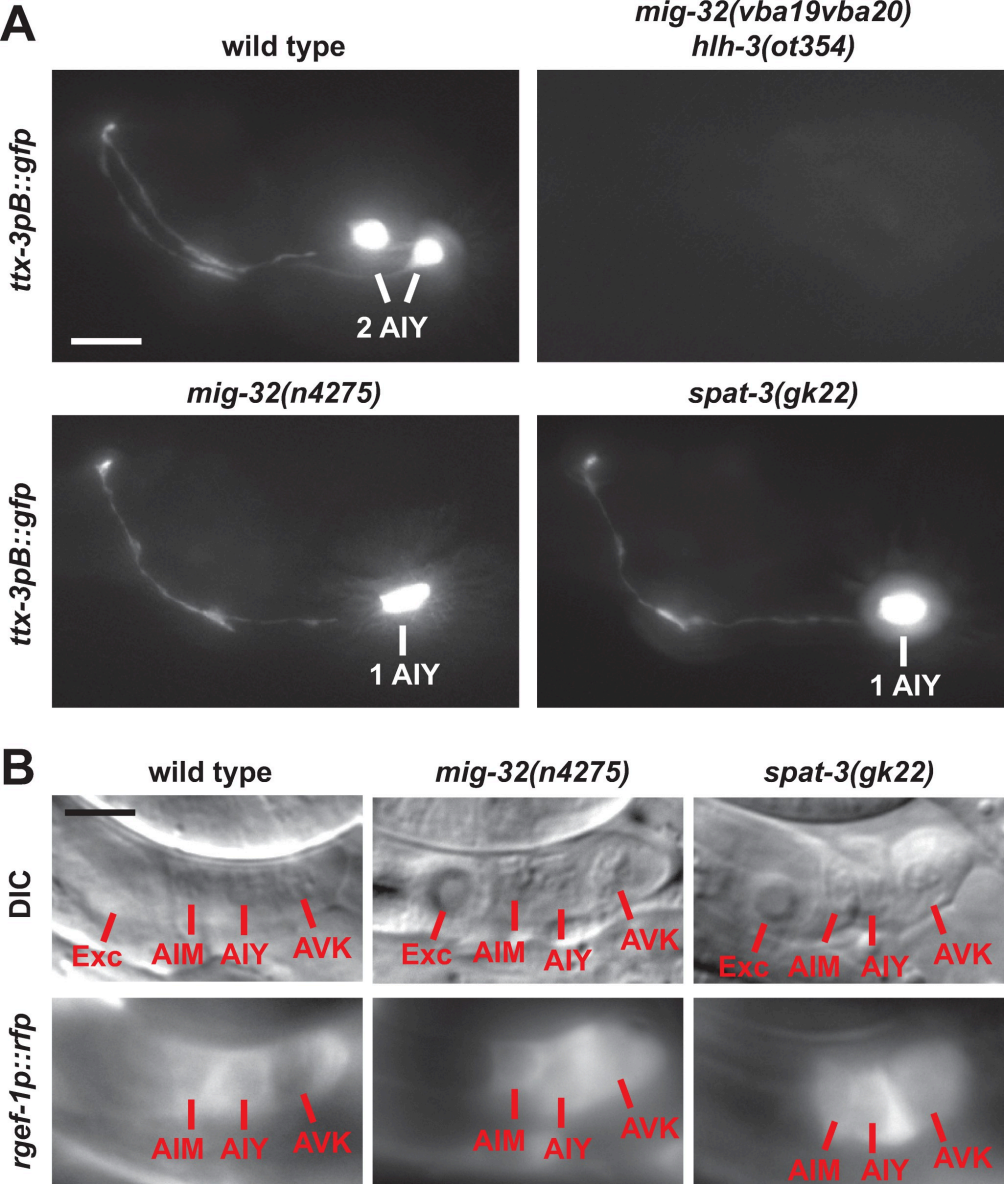
Effect of the PRC1 factors MIG-32 and SPAT-3 on the AIY neuron at larval stage. (A) Expression of *ttx-3pB*::*gfp* (*otIs173*) in the head of wild type or mutant L4 larvae (lateral view, anterior is left, dorsal is up, scale bar = 10μm). (B) The AIY neuron is located in a group of three neurons posterior to the excretory cell (Exc) and expresses the pan-neuronal marker *rgef-1* (*rgef-1p*::*dsRed2*, *otIs173*) in wild type L4 larvae. In *mig-32* and *spat-3* mutants, the AIY neurons that have lost *ttx-3pB*::*gfp* expression still express the pan-neuronal marker *rgef-1p*::*dsRed2* (5 AIY neurons with loss of *ttx-3pB*::*gfp* expression analyzed for each mutant genotype). Note that the *rgef-1p*::*dsRed2* signal is mostly cytoplasmic (lower in the nucleus), scale bar = 5 μm.

Via a genetic screen for regulators of AIY neuron fate, we isolated a mutant strain with a partially penetrant loss of the AIY marker *ttx-3pB*::*gfp* (transgene with *gfp* expression driven by a *cis*-regulatory element of the *ttx-3* gene, [13]) at L4 larval stage. We previously identified a mutation present in this strain, an early stop in the bHLH factor HLH-3 (*ot354*, a putative null allele) [16]. However, during backcrossing of the initially isolated mutant strain, we noticed a drop in the penetrance of the phenotype suggesting that the initial strain contains an additional mutation that enhances the phenotype. Using whole-genome sequencing, we compared the genome of strains segregating low or high penetrance AIY phenotypes, and identified that the enhancer mutation is in the *mig-32* gene (allele, *vba19vba20*). In *hlh-3(ot354)* single mutants, *ttx-3pB*::*gfp* expression is lost in about 40% of the animals, while in *hlh-3(ot354); mig-32(vba19vba20)* double mutants the penetrance increases to 80% (Figs 1B and 2A). *mig-32(vba19vba20)* also increases the penetrance of another allele of *hlh-3*, *tm1688* (a deletion in *hlh-3* and likely null allele).

MIG-32 is a core component of the PRC1 complex. It is the ortholog of the PCGF proteins of vertebrates (such as BMI1) and the Posterior sex combs (Psc) protein of *Drosophila* [22]. This protein family is characterized by a RING domain and a downstream conserved region. The *vba19vba20* allele contains two point mutations in *mig-32*. The first, *vba19*, converts a highly conserved Asparagine of the RING domain into a Tyrosine, and the second, *vba20*, changes a non-conserved Cysteine of the downstream region into a Tyrosine (Fig 1C). We confirmed the phenotype with a null allele of *mig-32*, *n4275* [22]. As observed with *mig-32(vba19vba20)*, *mig-32(n4275)* enhances the phenotype of *hlh-3* mutants (Fig 1B). Interestingly, while *mig-32(vba19vba20)* does not produce defects on its own, *mig-32(n4275)* alone induces a loss of *ttx-3pB*::*gfp* expression in about 15% of the animals, suggesting that *mig-32(vba19vba20)* is a hypomorphic allele (Figs 1B and 2A).

The core of the PRC1 complex is composed by the association of MIG-32 with another protein, SPAT-3 (ortholog of RING1 in vertebrates) [22]. We therefore analyzed the phenotype of a strong loss-of-function allele of *spat-3*, *gk22* [22]. *spat-3(gk22)* induces a loss of *ttx-3pB*::*gfp* expression in about 15% of the animals and enhances the phenotype of *hlh-3* mutants, which is similar to the effect observed with *mig-32(n4275)* (Figs 1B and 2A). In addition, the penetrance of *mig-32(n4275); spat-3(gk22)* double mutants is similar to the one of single *mig-32(n4275)* or *spat-3(gk22)* mutants in agreement with the fact that both proteins are required for the function of the PRC1 complex (Fig 1B).

PRC1 factors often work together with proteins of a second complex, PRC2, although cases where PRC1 acts independently of PRC2 have also been reported [21]. We therefore tested whether PRC2 factors are involved in the regulation of AIY fate. Mutations in the PRC2 core component genes (*mes-2*, *mes-3* and *mes-6*), which abolish PRC2 function [24–26], have no effect on *ttx-3pB*::*gfp* expression (S1A Fig). This suggests that the function of PRC1 in AIY fate is independent of PRC2.

### PRC1 factors regulate the type-specific identity of the AIY neuron

To better characterize the role of PRC1 factors in neuronal specification we first looked at the effect of PRC1 mutants on various features of the AIY neuron. In *mig-32(n4275)* and *spat-3(gk22)* loss of function mutants we observed, at L4 larval stage, a partially penetrant loss of the expression of AIY terminal differentiation markers: *sra-11* (a G protein-coupled receptor) and *inx-18* (an innexin) (S1B and S1C Fig). However, when the expression of AIY type identity markers is lost, the cell body of the AIY neuron is still present and this neuron still expresses the pan-neuronal marker *rgef-1* (Fig 2B). This suggests that PRC1 factors regulate the type-specific identity of the AIY neuron but not its pan-neuronal features, a phenotype similar to the one observed when the function of the terminal selector transcription factors TTX-3 and CEH-10 is lost [13,27]. However, one key difference is that a loss of function of TTX-3 or CEH-10 leads to a fully penetrant loss of AIY type-specific fate, while a loss of function of PRC1 only leads to a low penetrance phenotype. One possibility is that PRC1 factors could regulate AIY fate by rendering the expression of the key terminal selector transcription factors TTX-3 and CEH-10 more stable, increasing their resistance to intrinsic noise, genetic perturbations or environmental variations.

### PRC1 factors regulate the expression levels of the terminal selector transcription factors TTX-3 and CEH-10

**Fig 3:**
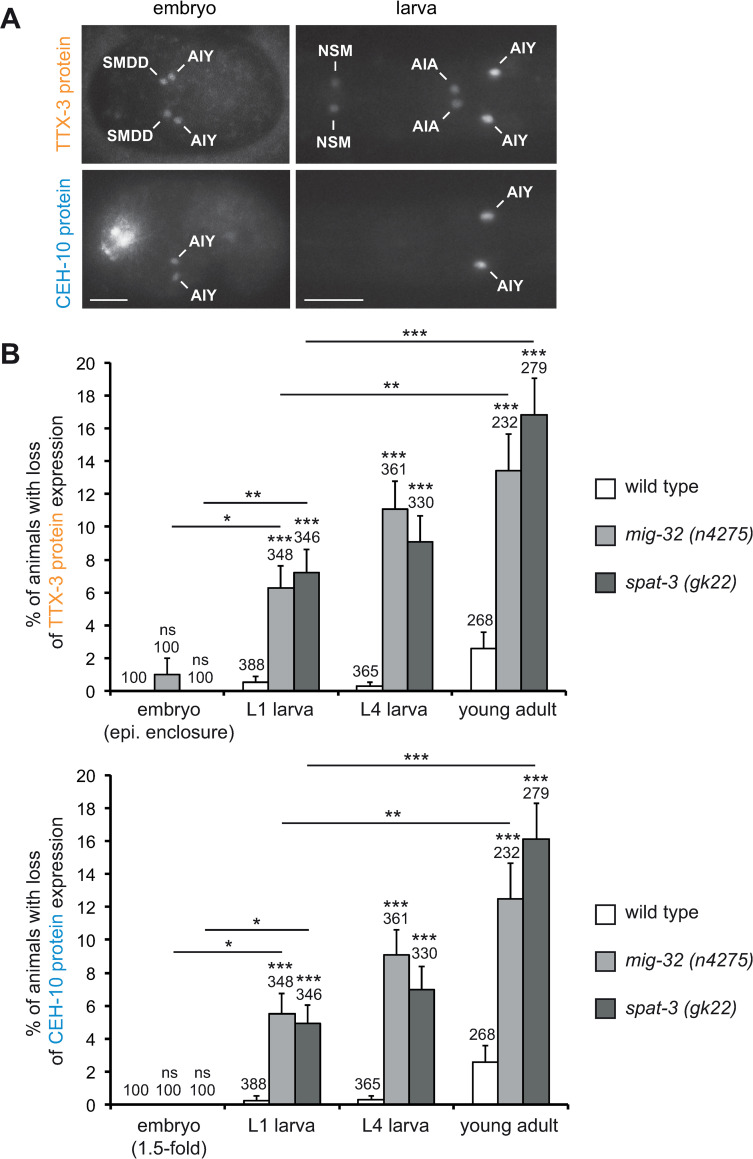
Progressive loss of TTX-3 and CEH-10 expression in PRC1 mutants. (A) Expression of the TTX-3 protein (*ttx-3endo*::*yfp (vba3)*) and the CEH-10 protein (*ceh-10endo*::*yfp (vba1)*) in embryos (epidermal enclosure for TTX-3, 1.5-fold for CEH-10) and early larvae. Ventral views, anterior is left, scale bar = 10 μm. (B) Percentage of animals that display a loss of TTX-3 protein (upper part) or CEH-10 protein (lower part) expression in at least one AIY neuron (error bars show standard error of proportion, numbers above the bars show number of animals analyzed). Expression in the embryo was analyzed at the stage when expression is initiated: epidermal enclosure for TTX-3 and 1.5-fold for CEH-10. For early larval (L1), late larval (L4) and young adult stages, TTX-3 and CEH-10 expressions were analyzed in parallel in the same animals using two color imaging. In most cases (86% of the neurons with a loss of expression) both TTX-3 and CEH-10 expression are lost. In the remaining cases (14%) only TTX-3 expression is lost while CEH-10 expression is still detectable. Statistical tests were performed between wild type and PRC1 mutants at each stage. They were also performed between embryonic and L1 larval stages, as well as between L1 larval and young adult stages in PRC1 mutants. A Fisher’s exact test was used (*** p<0.001, ** p<0.01, * p<0.05, ns not significant).

To quantify the effect of PRC1 mutants on the endogenous protein levels of the terminal selector transcription factors we used lines where the endogenous *ttx-3* and *ceh-10* genes were tagged in frame with YFP by CRISPR genome engineering at the endogenous loci (*ttx-3endo*::*yfp* and *ceh-10endo*::*yfp*) [17], and generated a line with the endogenous *ttx-3* gene tagged in frame with mKate2 (*ttx-3endo*::*mkate2*) (see Materials and Methods). The expression pattern observed is consistent with the expression previously described using transcriptional and translational reporters (Fig 3A) [15,27–29]. Using these lines, we then analyzed at what developmental stage the expression of TTX-3 and CEH-10 is lost in PRC1 mutants. In wild type animals, TTX-3 expression starts in the embryo during neurulation (epidermal enclosure) when it is detected in the newly generated AIY and SMDD postmitotic neurons (Fig 3A, [15]). In *mig-32* and *spat-3* mutants, TTX-3 expression is still present in the AIY and SMDD neurons at this stage (Fig 3B). CEH-10 expression starts in the postmitotic AIY neuron shortly after TTX-3 (during elongation, at the 1.5-fold stage of the embryo) (Fig 3A, [15]). In *mig-32* and *spat-3* mutants, CEH-10 expression is still detected in the AIY neuron at this stage (Fig 3B). This suggests that, in PRC1 mutants, TTX-3 and CEH-10 expression is still initiated in the AIY neuron during embryogenesis. We then analyzed the effect of PRC1 mutants on TTX-3 and CEH-10 maintenance at early larval (L1), late larval (L4) and early adult stages (one day old adult). We observed a progressive loss of both TTX-3 and CEH-10 expression as the animals get older, the percentage of animals displaying a loss of expression increasing with time (Fig 3B). This suggests that the specific type identity of the AIY neurons is progressively lost in a stochastic manner during the maintenance phase in PRC1 mutants.

**Fig 4:**
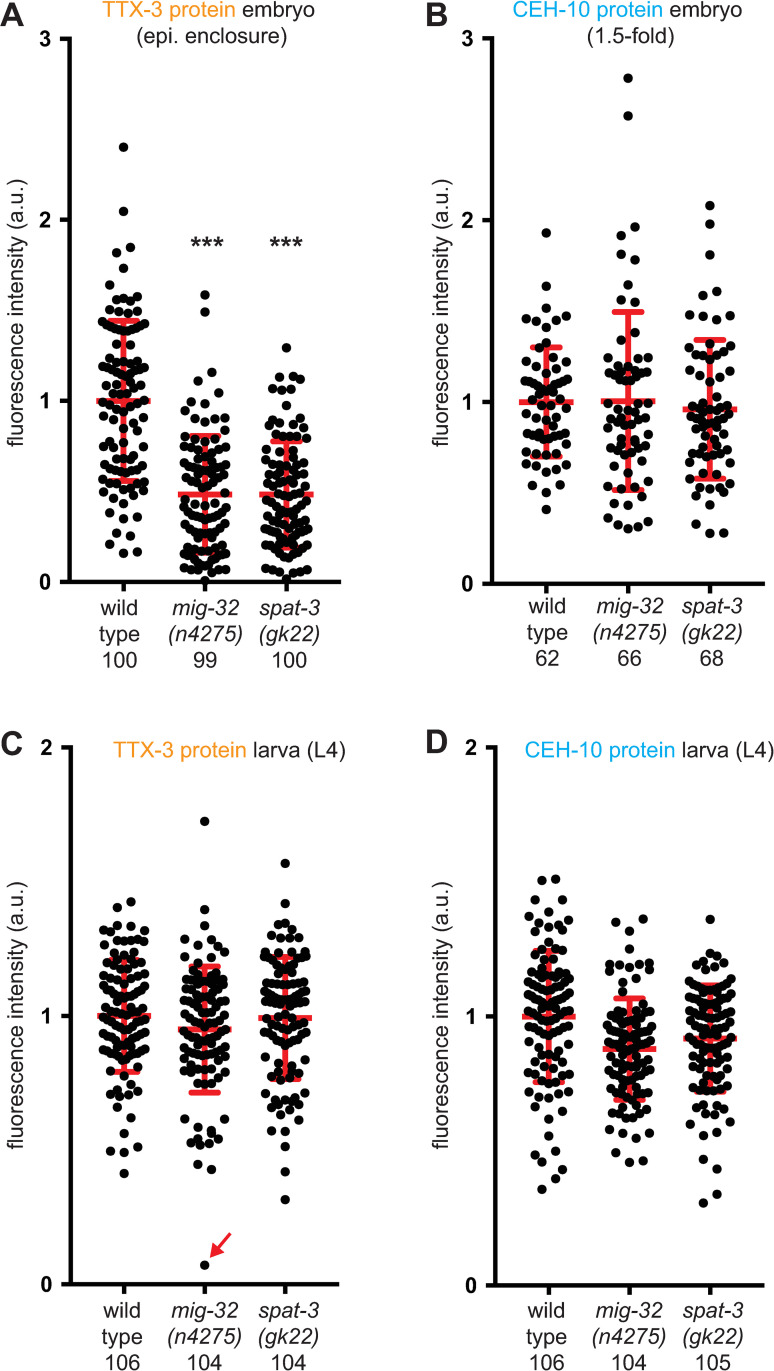
Effect of PRC1 mutants on TTX-3 and CEH-10 protein levels. Quantification of the fluorescence levels (arbitrary units) in AIY neurons of: (A) TTX-3 protein (*ttx-3endo*::*yfp (vba3)*) at epidermal enclosure embryonic stage; (B) CEH-10 protein (*ceh-10endo*::*yfp (vba1)*) at 1.5-fold embryonic stage; (C, D) TTX-3 protein (*ttx-3endo*::*mkate2 (vba6)*) and CEH-10 protein (*ceh-10endo*::*yfp (vba1)*) at L4 larval stage in the same neurons. Only neurons where expression was detected were analyzed. Each dot represents one neuron. The number of positive neurons analyzed for each condition (wild type, *mig-32(n4275)* or *spat-3(gk22)*) is presented below the genotype. The red bars represent the mean and SD. A Mann-Whitney U-test was used to compare the medians (*** p<0.001).

**Fig 5:**
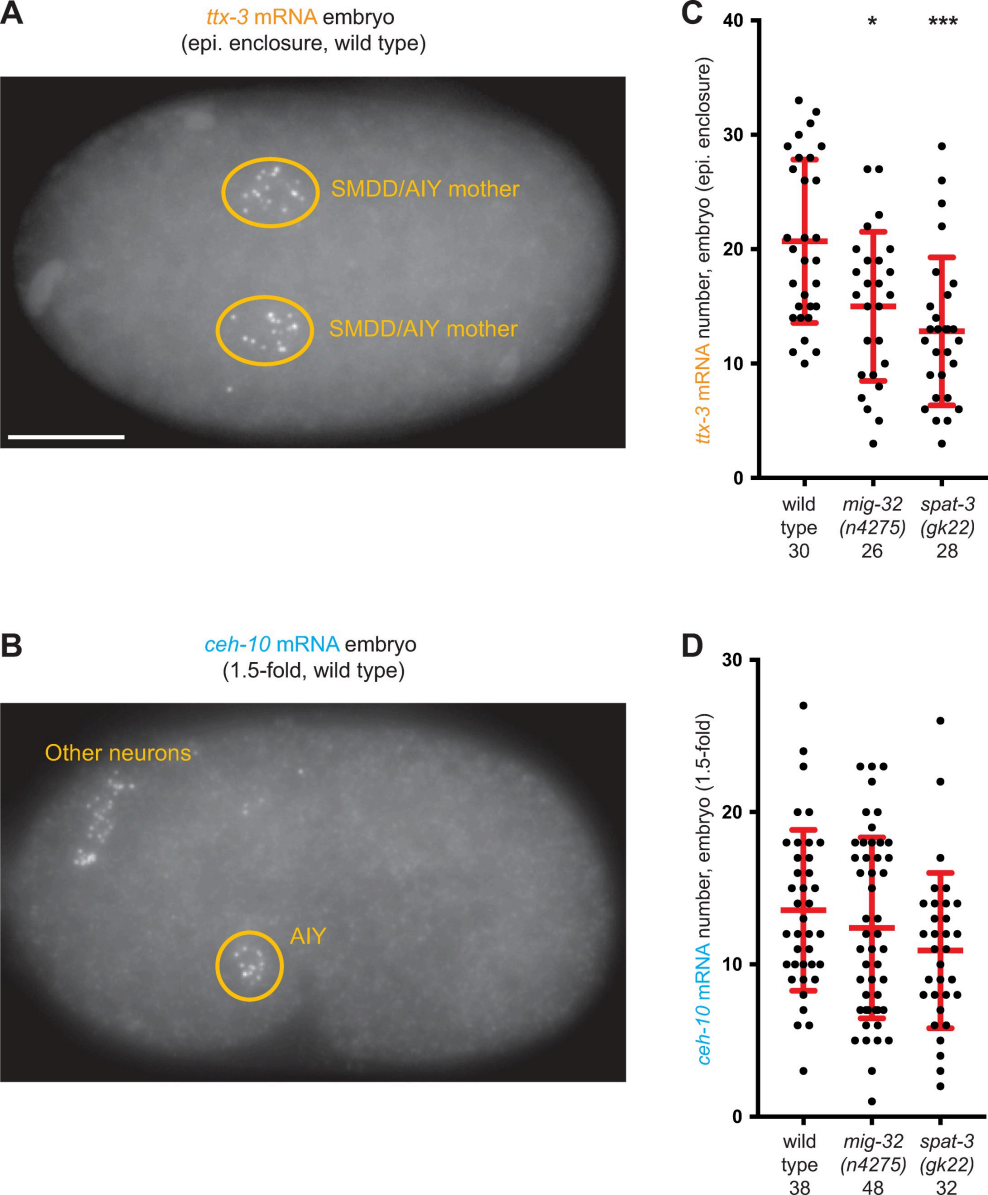
Effect of PRC1 mutants on *ttx-3* and *ceh-10* mRNA levels. (A) Detection of *ttx-3* mRNA by smFISH in a wild type embryo at epidermal enclosure stage. Due to the time delay induced by protein synthesis, the optimal time point for detection of *ttx-3* mRNA is in the SMDD/AIY mother cell, while the optimal time point for detection of TTX-3 proteins is a bit later in the early postmitotic AIY. Ventral view, anterior is left, scale bar = 10 μm. (B) Detection of *ceh-10* mRNA by smFISH in a wild type embryo at 1.5-fold stage. In addition to AIY in the ventral head, *ceh-10* mRNA are also present in a few other neurons in the dorsal head. Lateral view, anterior is left, dorsal is up. (C) Quantification of the number of *ttx-3* mRNA in the SMDD/AIY mother cells of wild type or PRC1 mutant embryos at epidermal enclosure stage. Each dot represents one cell. The number of cells analyzed for each condition (wild type, *mig-32(n4275)* or *spat-3(gk22)*) is presented below the genotype. The red bars represent the mean and SD. A Mann-Whitney U-test was used to compare the medians (*<0.05, *** p<0.001). (D) Quantification of the number of *ceh-10* mRNA in the AIY neurons of wild type or PRC1 mutant embryos at 1.5-fold stage.

We then analyzed whether mutations of PRC1 factors also affect the levels of expression of the TTX-3 and CEH-10 proteins when they are still detected in the AIY neurons. To this aim, we quantified the levels of endogenous TTX-3 and CEH-10 proteins using our CRISPR lines in both wild type and PRC1 mutant animals. In PRC1 mutants, we observed a decrease of the mean level of TTX-3 proteins during the initiation phase in the embryo (Fig 4A). However, we didn’t observe a change of the mean level of CEH-10 proteins during the initiation phase (Fig 4B). To determine whether the PRC1 complex affects *ttx-3* or *ceh-10* at the step of transcription, we quantified their mRNA numbers using smFISH (Fig 5A and 5B). We observed that PRC1 mutants decrease the mean number of *ttx-3* mRNA during the initiation phase in the embryo (Figs 5C and S2A), while they do not significantly affect *ceh-10* mRNA numbers (Figs 5D and S2B). These results are similar to the ones observed at protein level. Taken together, these data suggest that the expression of the terminal selector transcription factors TTX-3 and CEH-10 is still initiated in PRC1 mutants but the initiation level for TTX-3 is reduced.

During the maintenance phase, at late larval stage (L4), in PRC1 mutants, TTX-3 and CEH-10 protein expression is absent in some AIY neurons but not all (Fig 3B). We therefore analyzed the levels of expression of TTX-3 and CEH-10 proteins at this stage in AIY neurons that still expressed them (Fig 4C and 4D for data with neurons showing no expression excluded; S3 Fig for data with neurons showing no expression included). We did not observe a strong difference of levels between the AIY neurons that still express TTX-3 and CEH-10 in PRC1 mutants and the AIY neurons of wild type animals. Thus, in PRC1 mutants, while TTX-3 and CEH-10 expression is completely absent in some AIY neurons, their levels seem mostly unaffected in the remaining AIY neurons. This may be explained by the positive feedback loop that maintains TTX-3 and CEH-10 expression as such network motifs can lead to bistable ON-OFF states [5]. However, in PRC1 mutants, we noticed some very rare cases (less than 0.5%) of neurons with very low levels of TTX-3 or CEH-10 proteins (see for example Fig 4C, red arrow). They may represent neurons transitioning from the ON state to the OFF state.

In the embryo, the level of TTX-3 expression is reduced in PRC1 mutants during the initiation phase. As HLH-3 regulates the initiation of TTX-3 expression in the embryo, we tested whether HLH-3 expression may also be affected in PRC1 mutants. Using a line where the endogenous *hlh-3* gene is tagged in frame with mNeonGreen, we observed that HLH-3 expression is not affected in the AIY lineage of the embryo (epidermal enclosure stage) (S4 Fig). This suggests that the PRC1 complex acts in parallel with HLH-3.

### PRC1 factors act in the AIY lineage

**Fig 6:**
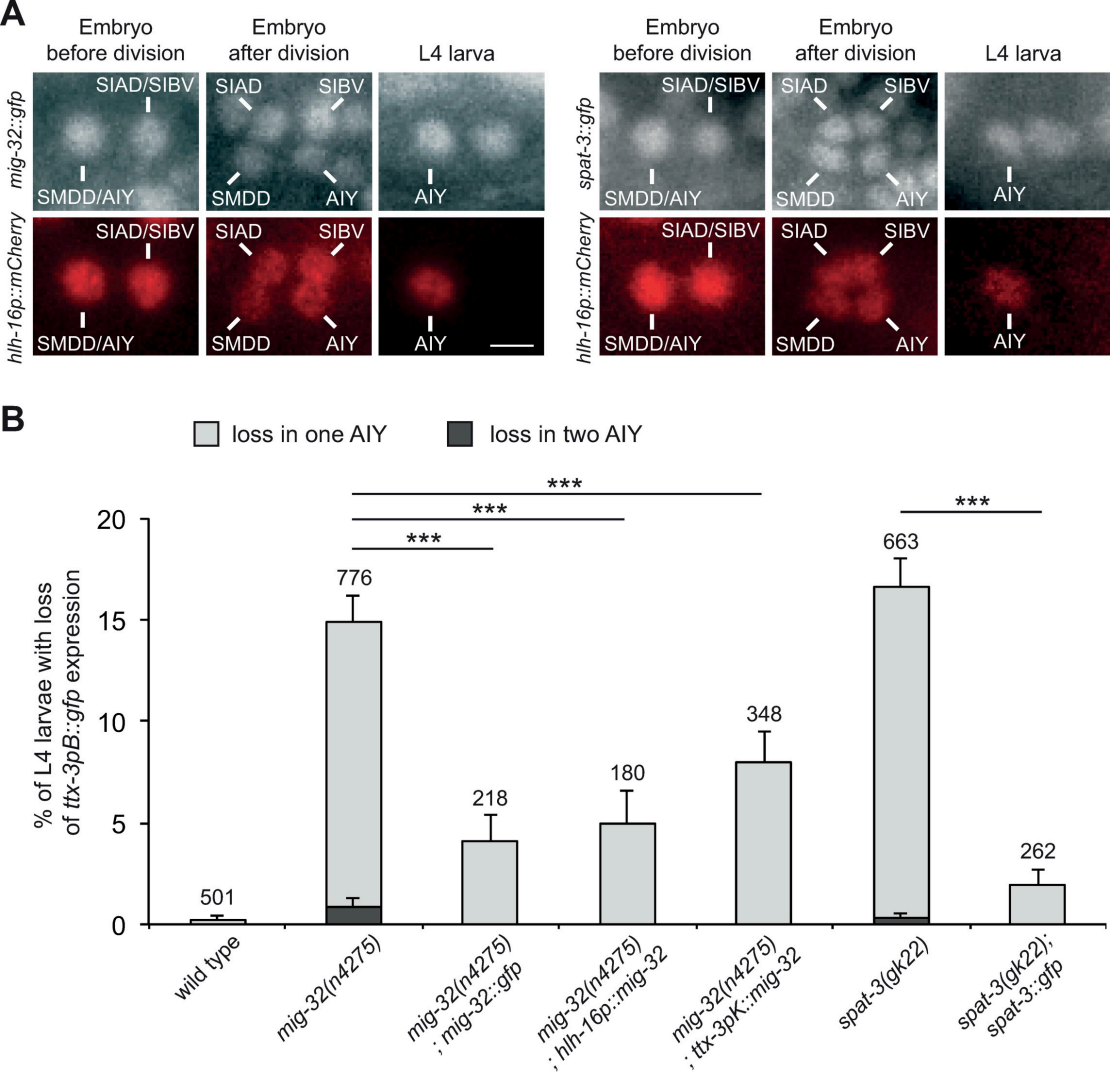
Expression of the PRC1 factors and cell-specific rescue. (A) Expression of *mig-32*::*gfp* (fosmid based translational fusion, *vbaIs44*) and *spat-3*::*gfp* (fosmid based translational fusion, *vbaIs47*) in the AIY lineage labeled with *hlh-16p*::*mCherry* (*hlh-16* promoter driving the expression of a nuclear mCherry). In the embryo, MIG-32 and SPAT-3 are detected in the SMDD/AIY mother cell and its sister cell, the SIAD/SIBV mother cell, before their terminal division. Following the terminal division, MIG-32 and SPAT-3 are detected in the newly generated SMDD, AIY, SIAD and SIBV neurons. In L4 larvae, MIG-32 and SPAT-3 are detected in the AIY neuron. Scale bar = 2 μm. (B) Percentage of L4 larvae that display a loss of *ttx-3pB*::*gfp* (*otIs173*) expression in one (grey bar) or two (black bar) AIY neurons (error bars show standard error of proportion, numbers above the bars show number of animals analyzed). The transgenes *mig-32*::*gfp* (fosmid based translational fusion, *vbaIs44*), *hlh-16p*::*mig-32* (*vbaEx158*), *ttx-3pK*::*mig-32* (*vbaEx155*) and *spat-3*::*gfp* (fosmid based translational fusion, *vbaEx127*) rescue the respective mutant (*** p<0.002, Fisher’s exact test).

To determine the site of action of the PRC1 factors we first analyzed their expression pattern. We generated lines expressing translational fusions of MIG-32 or SPAT-3 with GFP. GFP is inserted in frame just before the stop codon into fosmids containing the *mig-32* or *spat-3* locus as well as more than 18kb of upstream and 6kb of downstream sequences (covering also several upstream and downstream genes). These MIG-32::GFP and SPAT-3::GFP fusions rescue the loss of AIY fate in the respective *mig-32* and *spat-3* mutants (Fig 6B), suggesting that the fusion proteins are functional and that all the *cis*-regulatory elements necessary for their function in AIY fate determination are present.

MIG-32 and SPAT-3 are broadly expressed in many cells of the embryo, larvae and adults. They are enriched in the nucleus, as expected for chromatin factors. We observed expression of MIG-32 and SPAT-3 in the SMDD/AIY mother cell before its terminal division (Fig 6A). Following division, MIG-32 and SPAT-3 are detected in the postmitotic SMDD and AIY with similar levels between daughter cells. MIG-32 and SPAT-3 then remain expressed in the AIY neurons throughout embryogenesis, larval and adult stages.

To determine where PRC1 function is required, we performed cell type specific rescue of *mig-32* mutants. We first placed *mig-32* cDNA under the control of the *hlh-16* promoter, which drives expression first in the SMDD/AIY mother and then in the postmitotic AIY neuron throughout the life of the animal [30]. This promoter also drives expression in a few other neuronal lineages. This *hlh-16p*::*mig-32* construct rescues the loss of AIY fate observed at late larval stage (Fig 6B). We then placed *mig-32* cDNA under the control of the *ttx-3* promoter K (maintenance element), which drives expression only in the postmitotic AIY neuron during the maintenance phase (3-fold embryo to adult) [15]. This *ttx-3pK*::*mig-32* construct also significantly rescues the loss of AIY fate observed at late larval stage (Fig 6B). Taken together, these data suggest that the PRC1 complex directly acts in postmitotic AIY neurons to regulate their differentiated state. We noticed that the rescue is partial, which could be due to suboptimal expression levels of *mig-32* or contributions of other cell types in addition to AIY.

The PRC1 complex usually acts by ubiquitylating histone H2A on lysine 119 [19,20]. To test whether histone ubiquitylation is involved in AIY fate regulation by PRC1, we analyzed the effect of a point mutation in SPAT-3 that specifically affects histone ubiquitylation, *mgw14* [23]. In *spat-3(mgw14)* we observed a loss of AIY fate (S5 Fig) similar to the one observed in the loss-of-function mutants *spat-3(gk22)* and *mig-32(n4275)* (Fig 1B). This suggests that histone ubiquitylation is involved in the regulation of AIY fate by PRC1 factors.

### PRC1 factors protect the AIY neuron identity against stress

**Fig 7:**
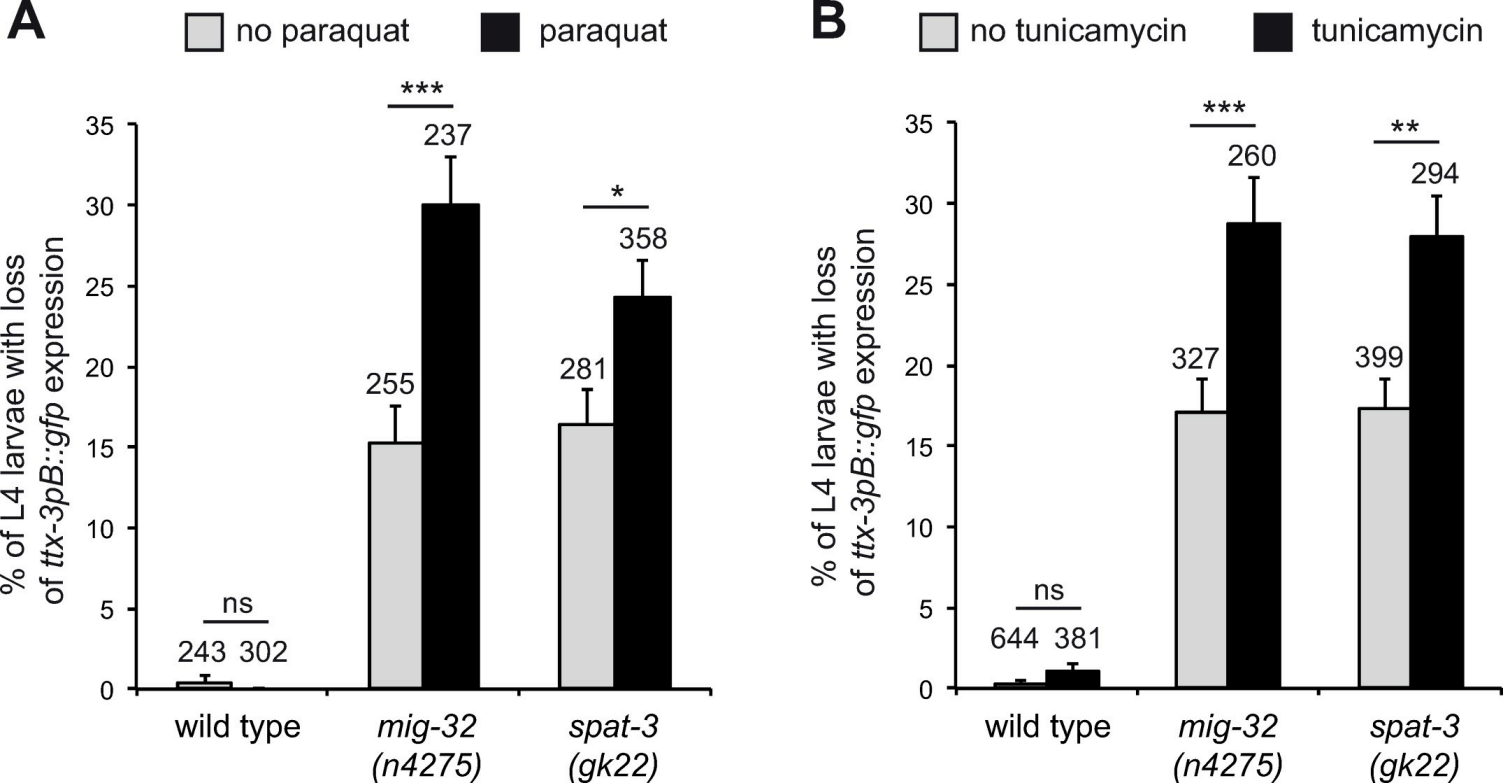
Effect of stress on AIY neurons. Percentage of L4 larvae that display a loss of *ttx-3pB*::*gfp* (*otIs173*) expression in one or two AIY neurons in wild type or PRC1 mutant animals, in absence or presence of paraquat (A) or tunicamycin (B). Error bars show standard error of proportion, numbers above the bars show number of animals analyzed (*** p<0.001, ** p<0.01, * p<0.05, ns not significant, Fisher’s exact test).

PRC1 factors are required for the reliable specification and maintenance of AIY specific identity under normal laboratory conditions. We next tested whether PRC1 factors could also help ensure the resistance of the AIY differentiation program against stressful environmental conditions. We analyzed the effects of oxidative stress (using paraquat) or Endoplasmic Reticulum (ER) stress (using tunicamycin) on AIY fate (Fig 7). While stress doesn’t affect the identity of the AIY neuron in wild type animals, we observed that, in *mig-32* and *spat-3* mutants, stress increases the loss of AIY fate. This suggests that PRC1 factors provide protection against stressful conditions. PRC1 factors are therefore needed to ensure the consistency of the AIY differentiation program, this role being more crucial under stressful conditions than under normal laboratory environment.

### Origin of the variability in the expression of the terminal selector transcription factors TTX-3 and CEH-10

**Fig 8:**
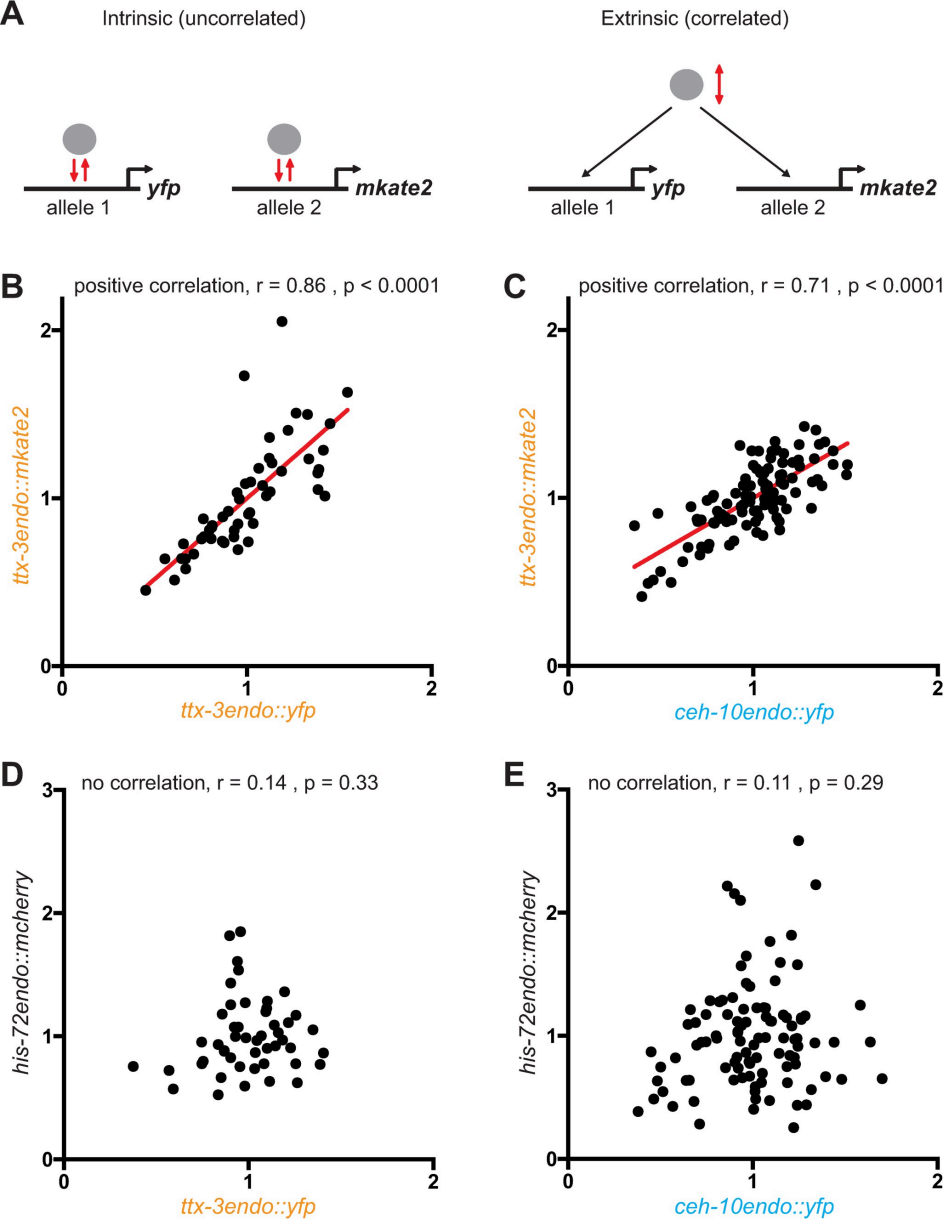
Characterization of the variability in *ttx-3* and *ceh-10* expression using correlation analysis. (A) Differentiation between intrinsic (uncorrelated) and extrinsic (correlated) sources of noise by tagging one allele of a gene with *yfp* and the other with *mkate2*. (B) Correlation between the fluorescence levels (arbitrary units) of *ttx-3endo*::*yfp* and *ttx-3endo*::*mkate2* (*vba3 / vba6* heterozygote animals, n = 52 neurons analyzed). (C) Correlation between the fluorescence levels of *ceh-10endo*::*yfp* and *ttx-3endo*::*mkate2* (*vba1; vba6* double homozygote animals, n = 106 neurons analyzed). (D) Correlation between the fluorescence levels of *ttx-3endo*::*yfp* and *his-72endo*::*mcherry* (*vba3; vba5* double homozygote animals, n = 49 neurons analyzed). (E) Correlation between the fluorescence levels of *ceh-10endo*::*yfp* and *his-72endo*::*mcherry* (*vba1; vba5* double homozygote animals, n = 101 neurons analyzed). Each grey dot represents one AIY neuron. r = Spearman’s correlation.

During our quantification experiments in wild type animals, we noticed variability in the levels of TTX-3 and CEH-10 proteins during both the initiation and maintenance phases (Fig 4). This suggests that there is noise in the genetic program that initiates and maintains the identity of the AIY neuron. While this variability doesn’t have phenotypic consequences in a wild type background (with a 100% accurate AIY differentiation), it could have deleterious effects in mutant backgrounds (such as PRC1 mutant backgrounds) contributing to the partial penetrance aspect of the phenotype. To better understand the origin of the variability of TTX-3 and CEH-10 levels we used a “two reporters” strategy initially developed in bacteria with transgenes [31] and subsequently applied to endogenous proteins tagged with fluorescent proteins in yeast [32]. We analyzed a strain where one allele of TTX-3 is tagged with YFP and the other allele with mKate2 (*ttx-3endo*::*yfp* / *ttx-3endo*::*mkate2*). We quantified at L4 larval stage the correlation between the proteins produced by both alleles in single AIY neurons (Fig 8A and 8B). This approach allows the differentiation between correlated (extrinsic) noise arising from variations in the concentration of common upstream regulators, from uncorrelated (intrinsic) noise that arises from stochasticity of the molecular interactions at a locus. We observed a good correlation between *ttx-3endo*::*yfp* and *ttx-3endo*::*mkate2* levels suggesting that most of the variability comes from noise in the levels of upstream regulators common to both alleles. However, the correlation is not perfect, suggesting that intrinsic noise, coming for example from the stochasticity of the transcription process at a single locus, also contribute to the variability. We also observed a significant correlation in single AIY neurons between *ttx-3endo*::*mkate2* and *ceh-10endo*::*yfp* levels (Fig 8C), indicating that part of the variability comes from noise in the levels of upstream regulators common to *ttx-3* and *ceh-10*. Finally, we did not observe any correlation in single AIY neurons between the levels of proteins of an unrelated housekeeping gene *his-72* and *ttx-3* or *ceh-10* (Fig 8D and 8E). This suggests that the variability in TTX-3 and CEH-10 proteins is not primarily coming from variability between cells in the global ability to produce proteins (that could come, for example, from different quantities of RNA polymerase II or ribosomes). Taken together, these data suggest that the variability in the levels of the terminal selector transcription factors comes from both intrinsic sources (locus specific variability in molecular interactions) and extrinsic sources (variability in upstream factor levels) with a stronger contribution of extrinsic sources.

### PRC1 is also implicated in the differentiation of other neuronal types

**Fig 9:**
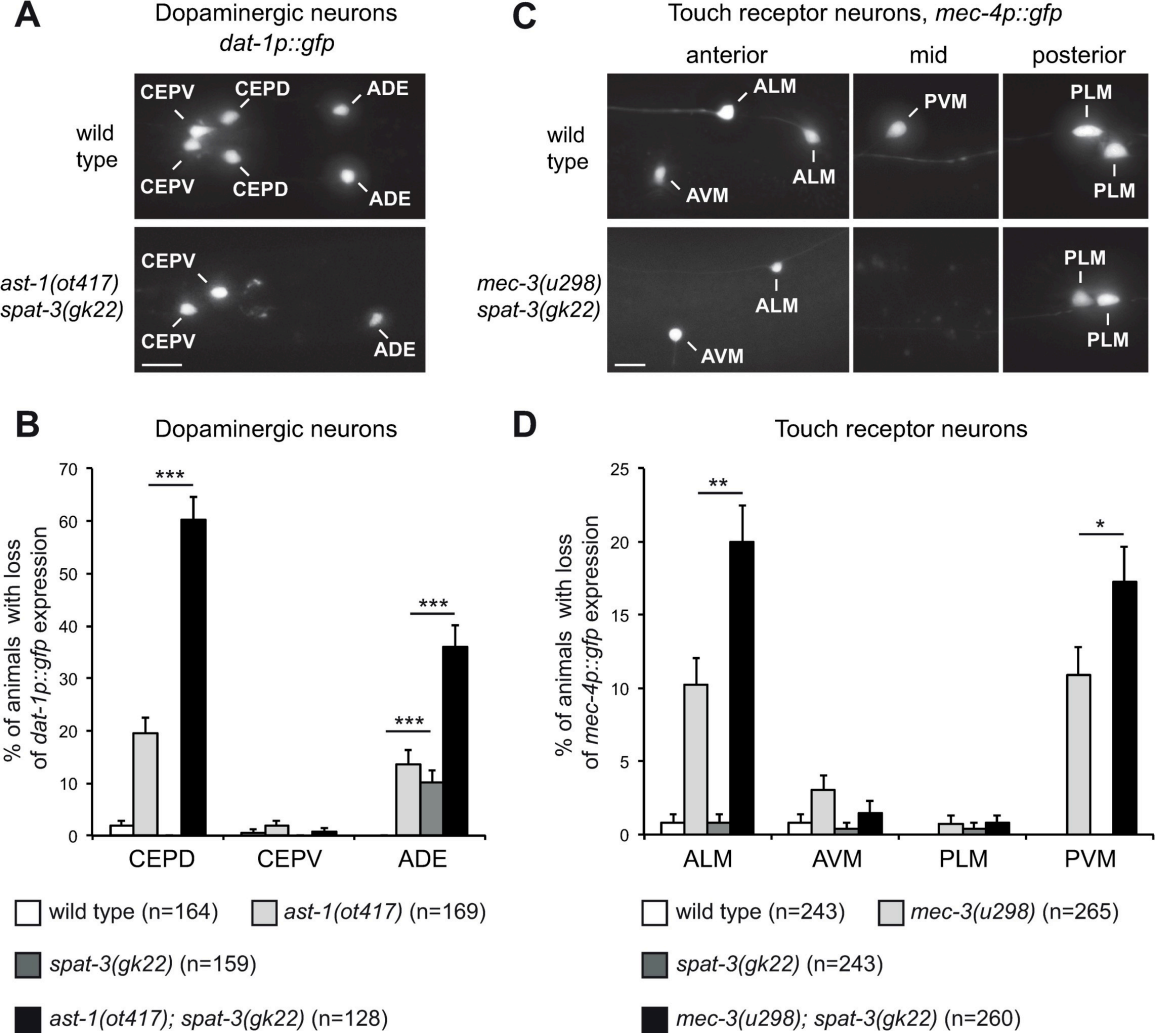
Effect of PRC1 on dopaminergic neurons and touch receptor neurons. (A) Expression of the dopaminergic marker (*dat-1p*::*gfp*, *vtIs1*) in the head of an early larva. In wild type animals expression is observed in the 6 dopaminergic neurons of the head (2 CEPV, 2 CEPD, 2 ADE). In *ast-1(ot417); spat-3(gk22)* double mutants expression is lost in some CEPD and ADE (expression lost in the two CEPD and one of the ADE in this example). Dorsal views, anterior is left, scale bar = 5 μm. (B) Percentage of early larvae that display for each class of head dopaminergic neurons a loss of *dat-1p*::*gfp* (*vtIs1*) expression in at least one neuron (error bars show standard error of proportion, n = number of animals analyzed, *** p<0.001, Fisher’s exact test). (C) Expression of the touch receptor neuron marker (*mec-4p*::*gfp*, *zdIs5*) in the anterior, mid and posterior regions of late larvae (L4). In wild type animals expression is observed in the 6 touch receptor neurons (2 ALM, 1 AVM, 2 PLM, 1 PVM). In *mec-3(u298); spat-3(gk22)* double mutants expression is lost in some ALM and PVM (expression lost in one of the two ALM and in PVM in these examples). Lateral views, anterior is left, scale bar = 5 μm. (D) Percentage of late larvae (L4) that display for each class of touch receptor neurons a loss of *mec-4p*::*gfp* (*zdIs5*) expression in at least one neuron (error bars show standard error of proportion, n = number of animals analyzed, * p<0.05, ** p<0.01 Fisher’s exact test).

We showed that the PRC1 complex affects the differentiation program of the AIY cholinergic interneuron. As PRC1 factors are also expressed in other neurons, we next asked whether the PRC1 complex also affects the identity of other neuronal types. We first looked at the effect on dopaminergic neuron fate. *C*. *elegans* larvae have 3 pairs of dopaminergic neurons in the head: CEPD, CEPV and ADE (Fig 9A). We observed that, in a loss of function of the PRC1 gene *spat-3*, the expression of the dopaminergic marker *dat-1* is lost in the ADE neuron in about 10% of the animals at early larval stage (Fig 9B). However, expression in CEPD or CEPV is unaffected. We next tested whether mutating PRC1 enhances the effect of a partial loss of function of a dopaminergic terminal selector transcription factor. The differentiation of the dopaminergic neurons is regulated by a terminal selector transcription factor of the ETS family, AST-1, that directly activates a large battery of dopaminergic differentiation genes including the dopamine transporter gene *dat-1* [33]. A partial loss of function of *ast-1* (the weak allele *ot417*) induces a partially penetrant loss of *dat-1* expression in CEPD and ADE (Fig 9B). Interestingly, a loss of function of the PRC1 gene *spat-3* strongly enhances this effect (Fig 9B). All together, this suggests that PRC1 also regulates the consistency of the differentiation program of dopaminergic neurons, and that the strength of the effect varies depending on the dopaminergic neuron analyzed.

We next characterized the effect of PRC1 on the touch receptor neurons. Late larvae of *C*. *elegans* have 6 touch receptor neurons: 2 ALM and 1 AVM in the anterior region; 1 PVM in the mid region; 2 PLM is the posterior region (Fig 9C). In a loss of function of the PRC1 gene *spat-3*, we observed no effect on the expression of touch receptor neuron marker *mec-4* at late larval stage (Fig 9D). We then tested whether the *spat-3* mutant enhances the effect of partial loss of function of a terminal selector transcription factor of touch receptor neurons. The differentiation of the touch receptor neurons is regulated by a terminal selector transcription factor of the homeodomain family, MEC-3, that directly activates a large battery of touch receptor differentiation genes including the ion channel gene *mec-4* [34]. A weak allele of *mec-3* (*u298*) induces a low penetrance loss of expression of the touch receptor neuron marker *mec-4* in the ALM and PVM neurons at late larval stage, and this loss is slightly but significantly enhanced in *spat-3* mutants (Fig 9D). This suggests that PRC1 also affects the consistency of the differentiation program of the ALM and PVM touch receptor neurons.

Taken together, these data suggest that the PRC1 complex affects the consistency of the differentiation of several neuronal types in *C*. *elegans*, and that the sensitivity of neurons to a loss of PRC1 varies depending on neuronal types.

## Discussion

In the nervous system, neurons need to acquire and maintain their differentiated type identity in an accurate manner. However, the mechanisms that ensure the precision of neuronal cell fate acquisition during development and maintenance throughout the life of the animal remain poorly characterized. Here we observed that PRC1 chromatin factors play a role in the consistency of neuronal fate specification and maintenance. PRC1 factors affect the precision of terminal selector expression levels during the initiation phase and the accuracy of their maintenance throughout the life of the neuron.

### Role of PRC1 factors during neuronal cell fate specification and maintenance in *C*. *elegans*

Previous studies have shown that PRC1 factors affect neuronal cell migration and axon guidance [22,23]. Here we show that PRC1 factors also affect the terminal differentiation of different classes of neurons in *C*. *elegans*. We analyzed the effect on the AIY cholinergic interneuron in more detail. In PRC1 mutants, during the initiation phase in the embryo, the terminal selector transcription factors, TTX-3 and CEH-10, are still expressed in the AIY neuron. However, the numbers of *ttx-3* mRNA and proteins are reduced. We have previously shown that the proneural bHLH factor HLH-3 directly contributes to the initiation of *ttx-3* expression by binding to E-boxes in its *cis*-regulatory regions [16]. The strong enhancement of AIY fate loss, which we observed when PRC1 mutants are combined with *hlh-3* mutants, may therefore come from their parallel positive role on the activation of *ttx-3* expression. During the maintenance phase, in larvae and adults, we observed a stochastic loss of TTX-3 and CEH-10 expression over time in PRC1 mutants. Quantification of TTX-3 and CEH-10 expression levels in PRC1 mutants during the maintenance phase shows that it is essentially bimodal with most neurons showing either high wild type levels or no expression at all. It has been shown that positive feedback loops can generate bistable ON-OFF states [5], therefore the positive feedback loop that maintains TTX-3 and CEH-10 expression [13] is likely the cause of this bimodal distribution. The rare neurons that we observed with intermediate levels may represent neurons that transition from the ON state to the OFF state.

We observed that PRC1 factors act cell autonomously in the AIY neuron and that their activity seems continuously required, as restoration of PRC1 activity during the maintenance phase partially rescues the loss of AIY fate. How are PRC1 factors acting on *ttx-3* and *ceh-10* transcription? While PRC1 factors have been initially characterized as repressors of gene transcription, it was more recently shown that they can also act in a positive manner on the transcription of their direct targets [19–21]. PRC1 factors could therefore directly promote the transcription of *ttx-3* and *ceh-10*. For example, it has been observed that PRC1 factors can help to prime genes for future expression via the establishment of bivalent domains with both activation and repressive chromatin marks [19,21]. This priming mechanism allows a more efficient transcriptional activation at later stages and could explain, for example, the effect that we observed on the initiation of *ttx-3* expression. It has also been shown that PRC1 factors can have a positive role on transcription by helping the interaction between enhancers and promoters [35]. PRC1 factors may therefore help to ensure an accurate expression of *ttx-3* and *ceh-10* by securing the interaction between the promoter and the enhancer responsible for expression in AIY. PRC1 factors could also have a positive role on *ttx-3* and *ceh-10* in an indirect manner, by repressing the expression of potentially deleterious genes such as transcription factors specifying other fates. Interestingly, it has been observed that, in striatal neurons of mice, loss of the other Polycomb complex, PRC2, leads to the ectopic expression of transcription factors of other fates, potentially interfering with the striatal neuron differentiation program [36]. It would be interesting in the future to test whether this phenomenon also occurs in PRC1 mutants in mice.

### Variability and precision in neuronal cell fate specification and maintenance

By quantifying the mRNA and protein levels of the terminal selectors TTX-3 and CEH-10 in single AIY neurons, we observed variability of their expression level between different AIY neurons for both mRNA and proteins. This suggests that there is noise in their expression. This variability is present both during the initiation phase and during the maintenance phase. Interestingly, a similar variability was previously observed for the terminal selector gene *mec-3* in touch receptor neurons at mRNA level [37]. This suggests that the expression of terminal selectors may be noisy in general. To determine the origin of this variability, we performed correlation analysis in AIY neurons. Our data suggest that the variability in terminal selector expression mostly comes from extrinsic noise (variability in the level of upstream regulators) with a smaller contribution of intrinsic noise (stochasticity of molecular interactions at the locus). In addition, it was recently observed that the expression of terminal selectors can fluctuate over time as illustrated by CHE-1 expression in ASE gustatory neurons [38] or TTX-3 expression in AIY neurons [39]. In the AIY neurons, the noise in terminal selector expression doesn’t have phenotypic consequences in a wild type genetic background, where AIY differentiation is 100% accurate. This noise could have deleterious effects in PRC1 mutant backgrounds contributing to the partial penetrance of the phenotype, although further investigations would be required to test this hypothesis.

Noise in gene expression can be a problem in deterministic circuits such as the programs that specify and maintain neuronal identity [5]. Indeed, specific mechanisms are present to reduce noise in the expression of key transcription factors or to limit the adverse effects of their variability, such as partial redundancy between network components [5,40,41]. For the terminal selectors in neurons, it has been observed that a cooperation with other types of transcription factors, called “guarantors” can limit the variability in their expression [37,42]. In touch receptor neurons, the Aristaless/ARX ortholog ALR-1 reduces the variability of the terminal selector gene *mec-3* expression level during the maintenance phase, ensuring that the differentiation of the touch receptor neurons is robust [37]. In addition, during the initiation phase, several homeodomain proteins cooperate to ensure an efficient activation of *mec-3* expression [42]. While the loss of function of terminal selector transcription factors leads to a 100% penetrant loss of neuron fate, the loss of function of guarantor transcription factors leads only to a low penetrance loss of neuron fate. Here we propose that a chromatin modifier complex also contributes to the consistency of terminal selector transcription factor expression, acting as a guarantor: in the AIY interneuron, PRC1 factors help to set the correct level of expression of the terminal selector *ttx-3* during the initiation phase, and ensure the reliability of terminal selector expression maintenance at later stages (S6 Fig). As PRC1 factors have also been implicated in nervous system development in vertebrates and neurodevelopmental disorders in human [43,44], it would be interesting, in the future, to analyze whether PRC1 factors play a role in the consistency of expression of key neuronal terminal selector transcription factors in vertebrates as observed here in *C*. *elegans*.

## Materials and methods

### Genetics

The *vba19vba20* allele was isolated in an EMS genetic screen using a worm sorter to select mutants with a loss of expression of the *ttx-3pB*::*gfp* reporter [15, 45]. Variants in an enhanced and a non-enhanced backcrossed versions were identified by whole-genome sequencing using the MAQGene analysis software as described [45,46].

### Expression constructs and transgenic strains

The *ttx-3endo*::*mkate2 (vba6)* line was generated by CRISPR using a *rol-6* co-conversion strategy [47]. The coding sequence of *mkate2* was inserted just before the stop codon of *ttx-3* with a SGGGGS linker. The sequence targeted by the guide RNA was: CAGAGGTGGTGTGTTGAGCTGG (PAM underlined). The CRISPR strain was backcrossed 3 times before analysis.

Fosmids, where the MIG-32 and SPAT-3 proteins are tagged in frame with GFP, were obtained from the TransgeneOme project (M. Sarov) [48]. They were injected at 50 ng/μL with 80 ng/μL of pRF4 coinjection marker to obtain extrachromosomal arrays. The arrays were subsequently integrated in the genome using X-ray irradiation and backcrossed 3 times before analysis.

The hlh-16p::mig-32 construct was generated by replacing in the hlh-16prom(-514)::gfp vector [30] the coding sequence of *gfp* by the *mig-32* cDNA. It was injected at 50 ng/μL with 80 ng/μL of pRF4 coinjection marker. The ttx-3pK::mig-32 construct was generated by replacing in the ttx-3promK::gfp vector [13] the coding sequence of *gfp* by the *mig-32* cDNA. It was injected at 10 ng/μL with 80 ng/μL of pRF4 coinjection marker.

### Stress assays

For the stress assays, L4 larvae were exposed to 0.6 mM of paraquat (Sigma-Aldrich) or 3.5 μg/ml of tunicamycin (Sigma-Aldrich) on NGM plates seeded with OP50 bacteria. When reaching adulthood, they were transferred to new plates with paraquat or tunicamycin, and left to lay eggs for 2 hours. The adults were then removed from the plates and their progeny was scored when reaching L4 stage.

### Imaging

Standard observations were performed under a Zeiss Axioplan 2 epifluorescence microscope equipped with a Zeiss AxioCam MRm camera and the AxioVision software.

Quantifications of fluorescence levels in *ttx-3endo*, *ceh-10endo* and *hlh-3endo* CRISPR lines were performed on a Nikon Eclipse Ti microscope equipped with a spinning disc module (Yokogawa CSU-X1), 515 nm / 561 nm lasers, an EMCCD camera (Photometrics Evolve), and the Metamorph software. Embryos or L4 larvae were mounted on a 2% agar pad between a slide and a coverslip. After 3D image stacks acquisition, fluorescence intensity inside the nucleus of the AIY neurons was measured using the Fiji software and corrected for background autofluorescence. At L4 larval stage we observed a positive correlation between the fluorescence intensity and the depth of the neuron (z position). We therefore corrected the intensity attenuation due to the depth of the neuron using an exponential fit [49]. At embryonic stages (epidermal enclosure and 1.5-fold) the z position of the neuron varies less and we did not observed any significant correlation between the fluorescence intensity and the depth of the neuron; we therefore did not apply any z correction for embryonic stages.

### smFISH

smFISH on *ttx-3* and *ceh-10* mRNA in embryos was performed on slides following a previously developed protocol [50] with few changes. After permeabilization by freeze-craking and fixation, embryos were hybridized overnight at 37°C in hybridization buffer (10% formamide, 2x SSC, 100 mg/mL dextran sulfate, 1 mg/mL *E*. *coli* tRNA, 10 mM vanadoribosyl complex, 0.2 mg/mL BSA) containing 0.125 μM of smFISH probes coupled with Quasar 670 (Stellaris). Samples were washed twice in 10% formamide / 2x SSC buffer (30 minutes incubation at 37°C for the second wash), then washed twice in 2x SSC buffer before being mounted in Vectashield Mounting Medium. Imaging was performed using a Nikon Ti2E microscope equiped with a 100x 1.45 NA objective and an Andor Ikon-M high sensitivity camera. Quantification of the mRNA numbers was performed using a previously developed MATLAB script [51]. The AIY neuron and SMDD/AIY mother cell were identified by their stereotyped position.

### Scoring the proportion of animals positive for fluorescent reporter expression

For the proportion of animals positive for fluorescent reporter expression under normal laboratory conditions, for each strain scoring was done in at least two sessions. For the test of perturbed environmental conditions (paraquat, tunicamycin), experiments were performed in triplicate.

### Statistics

Statistical analysis were performed using GraphPad Prism. Comparisons of proportions between wild type and mutant animals were performed using the non-parametric Fisher’s exact test (two-tailed). Comparisons of fluorescence levels between wild type and mutant animals were performed using the non-parametric Mann-Whitney U test (two-tailed). Correlations between fluorescence levels at two wavelengths were performed using Spearman’s correlation. The exact number of animals or neurons analyzed as well as the test used and p-value obtained are presented in the figures and figure legends.

## Supporting information

S1 Fig Effect of PRC1 and PRC2 mutants on AIY neuron markers. (A) Percentage of L4 larvae that display a loss of *ttx-3pB*::*gfp* (*otIs173*) expression in one or two AIY neurons in PRC2 mutant animals. (B) Percentage of L4 larvae that display a loss of *inx-18p*::*gfp* (*otIs182*) expression in one or two AIY neurons in wild type or PRC1 mutant animals. (C) Percentage of L4 larvae that display a loss of *sra-11p*::*gfp* (*otIs123*) expression in one or two AIY neurons in wild type or PRC1 mutant animals. *** p<0.001, Fisher’s exact test.
(TIF)

S2 Fig Images of *ttx-3* and *ceh-10* mRNA detection by smFISH (related to Fig 5). (A) Detection in the SMDD/AIY mother cell of *ttx-3* mRNA by smFISH in wild type or PRC1 mutant backgrounds at epidermal enclosure stage. Scale bar = 2 μm. (B) Detection in the AIY neuron of *ceh-10* mRNA by smFISH in wild type or PRC1 mutant backgrounds at 1.5-fold stage.
(TIF)

S3 Fig Effect of PRC1 mutants on TTX-3 and CEH-10 protein levels at larval stage with negative neurons added (related to Fig 4C and 4D). Same data and presentation as Fig 4C and 4D but with the addition of neurons where no expression was detected.
(TIF)

S4 Fig Effect of PRC1 mutants on HLH-3 protein levels. Quantification of the fluorescence levels (arbitrary units) in AIY neurons of HLH-3 protein (*hlh-3endo*::*mNeonGreen (vlc28)*) at epidermal enclosure embryonic stage. Each dot represents one neuron. The number of neurons analyzed for each condition (wild type, *mig-32(n4275)* or *spat-3(gk22)*) is presented below the genotype. The red bars represent the mean and SD.
(TIF)

S5 Fig Effect on AIY neurons of a point mutation of SPAT-3 that affects histone ubiquitylation. Percentage of L4 larvae that display a loss of *ttx-3pB*::*gfp* (*otIs173*) expression in one (grey bar) or two (black bar) AIY neurons (error bars show standard error of proportion, numbers above the bars show number of animals analyzed).
(TIF)

S6 Fig Effect of PRC1 on the specification and differentiation program of the AIY neuron. PRC1 factors help to set the correct level of *ttx-3* expression during the initiation phase, and ensure the consistency of *ttx-3* and *ceh-10* expression during the maintenance phase.
(TIF)

S1 Data Numerical data. (XLSX)
