# Supplementary figures and images for: PRC1 chromatin factors strengthen the consistency of neuronal cell fate specification and maintenance in C. elegans
Source: PLoS Genet. 2022 May 23;18(5):e1010209. doi: 10.1371/journal.pgen.1010209 (PMC9126393; doi:10.1371/journal.pgen.1010209)

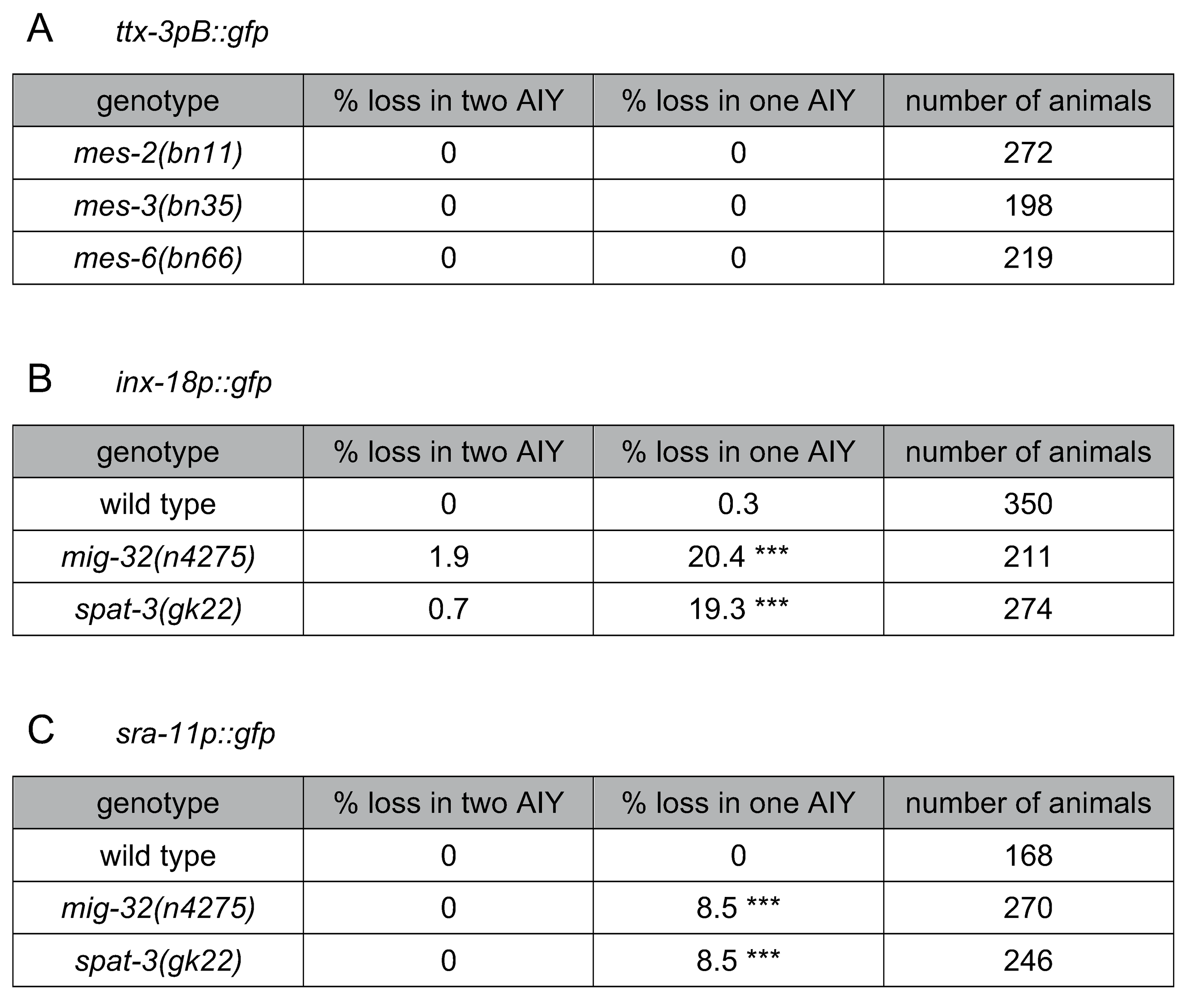

Supplement: S1 Fig — (A) Percentage of L4 larvae that display a loss of ttx-3pB::gfp (otIs173) expression in one or two AIY neurons in PRC2 mutant animals. (B) Percentage of L4 larvae that display a loss of inx-18p::gfp (otIs182) expression in one or two AIY neurons in wild type or PRC1 mutant animals. (C) Percentage of L4 larvae that display a loss of sra-11p::gfp (otIs123) expression in one or two AIY neurons in wild type or PRC1 mutant animals. *** p<0.001, Fisher’s exact test. (TIF) [file pgen.1010209.s001.tif]

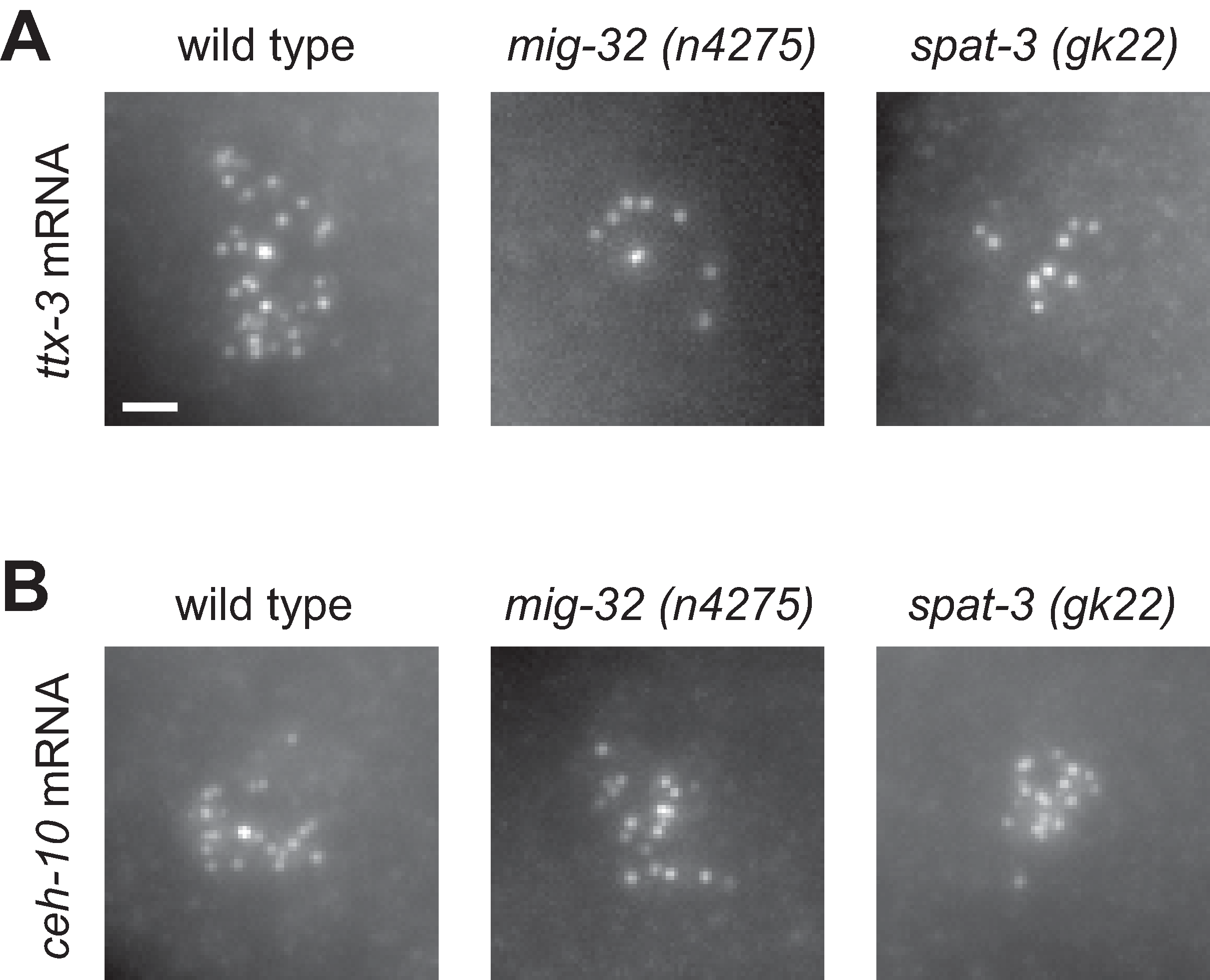

Supplement: S2 Fig — (A) Detection in the SMDD/AIY mother cell of ttx-3 mRNA by smFISH in wild type or PRC1 mutant backgrounds at epidermal enclosure stage. Scale bar = 2 μm. (B) Detection in the AIY neuron of ceh-10 mRNA by smFISH in wild type or PRC1 mutant backgrounds at 1.5-fold stage. (TIF) [file pgen.1010209.s002.tif]

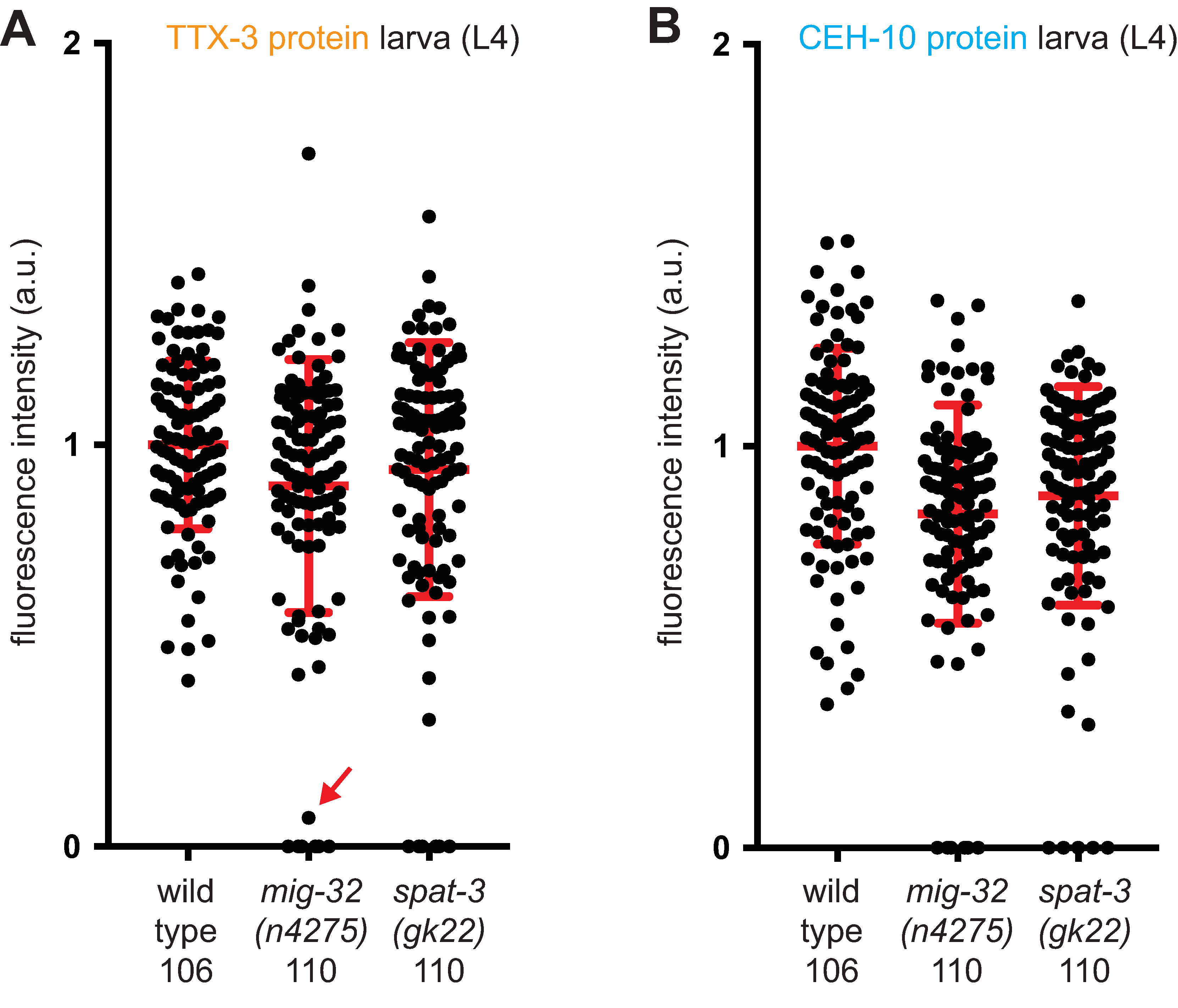

Supplement: S3 Fig — Same data and presentation as Fig 4C and 4D but with the addition of neurons where no expression was detected. (TIF) [file pgen.1010209.s003.tif]

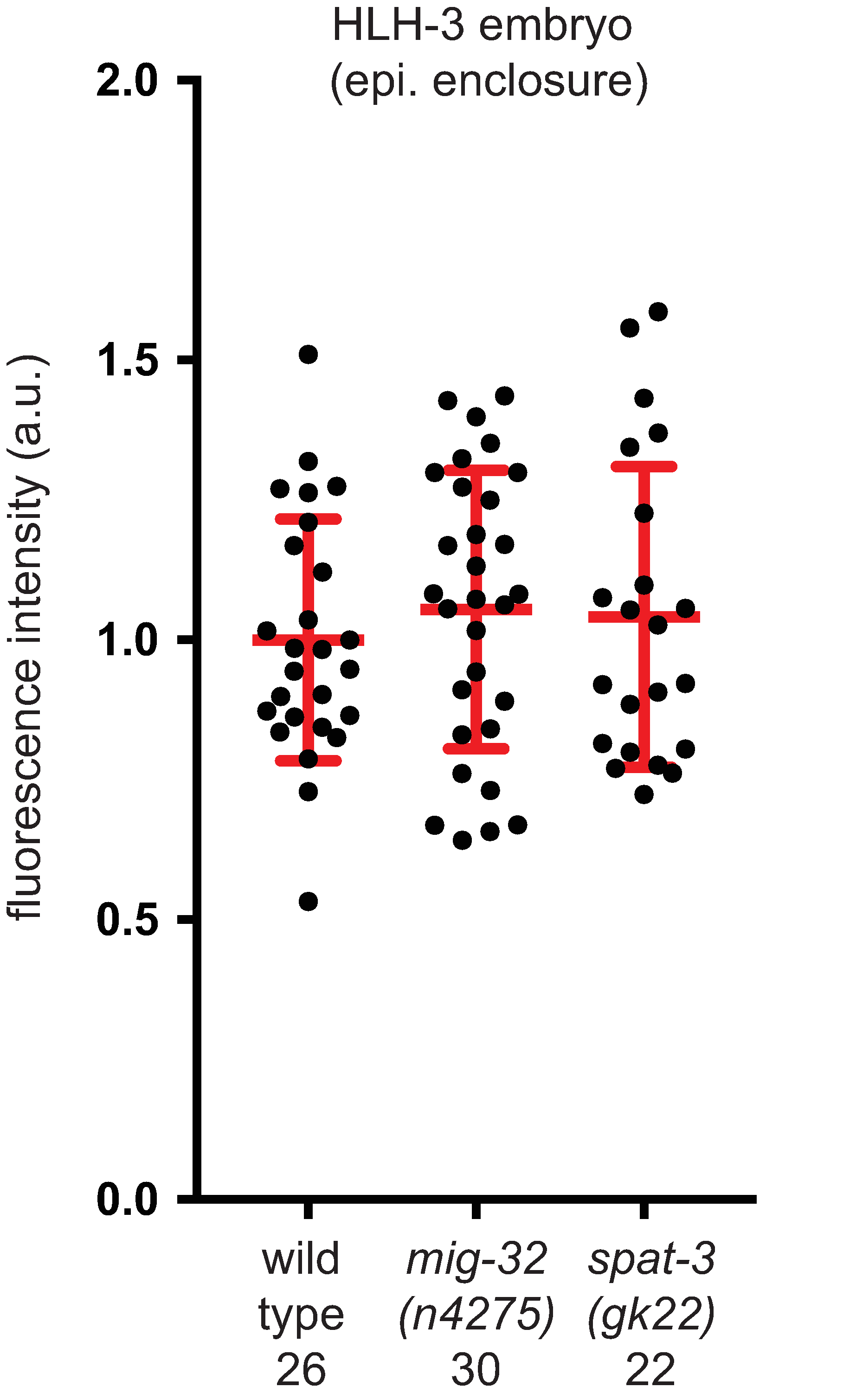

Supplement: S4 Fig — Quantification of the fluorescence levels (arbitrary units) in AIY neurons of HLH-3 protein (hlh-3endo::mNeonGreen (vlc28)) at epidermal enclosure embryonic stage. Each dot represents one neuron. The number of neurons analyzed for each condition (wild type, mig-32(n4275) or spat-3(gk22)) is presented below the genotype. The red bars represent the mean and SD. (TIF) [file pgen.1010209.s004.tif]

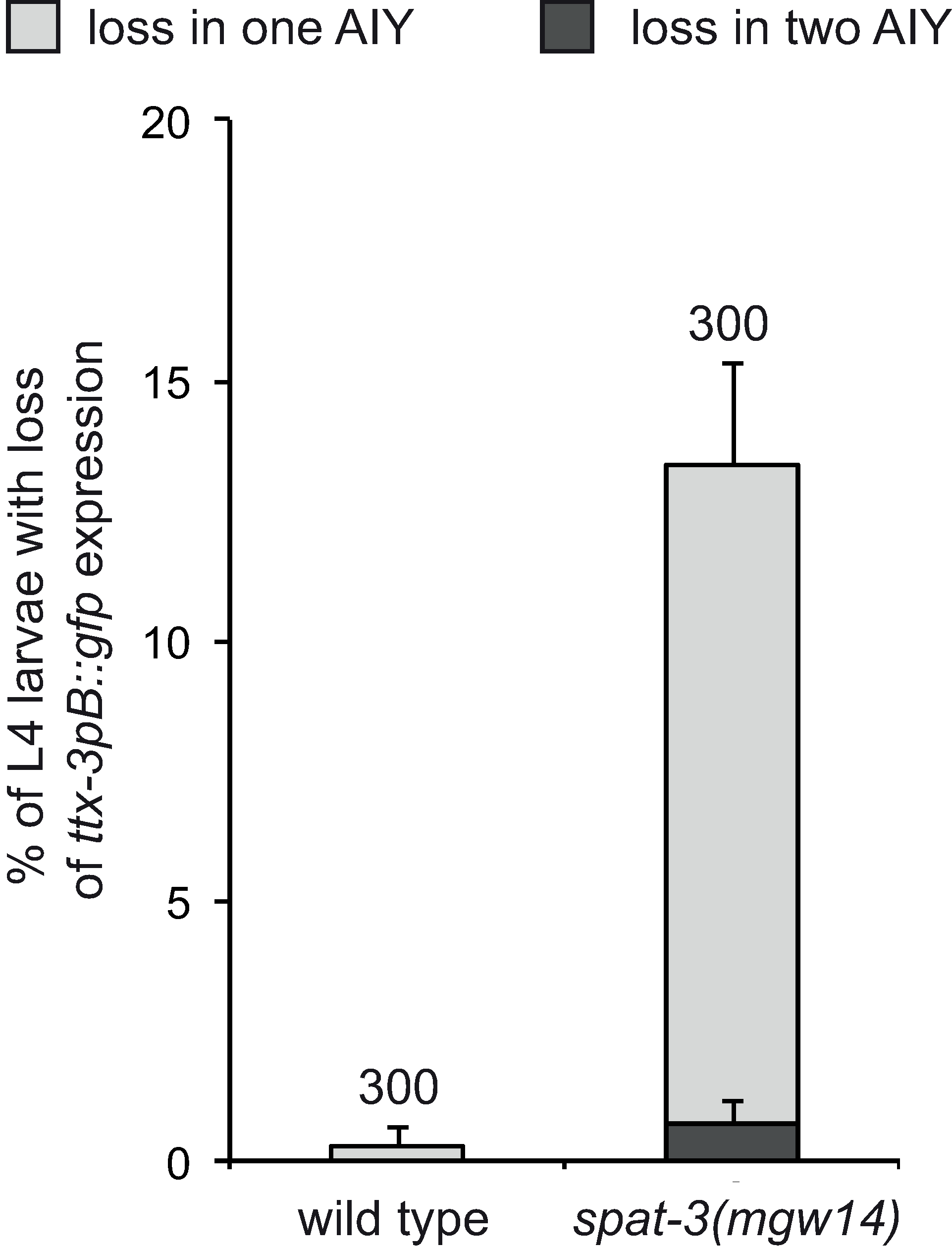

Supplement: S5 Fig — Percentage of L4 larvae that display a loss of ttx-3pB::gfp (otIs173) expression in one (grey bar) or two (black bar) AIY neurons (error bars show standard error of proportion, numbers above the bars show number of animals analyzed). (TIF) [file pgen.1010209.s005.tif]

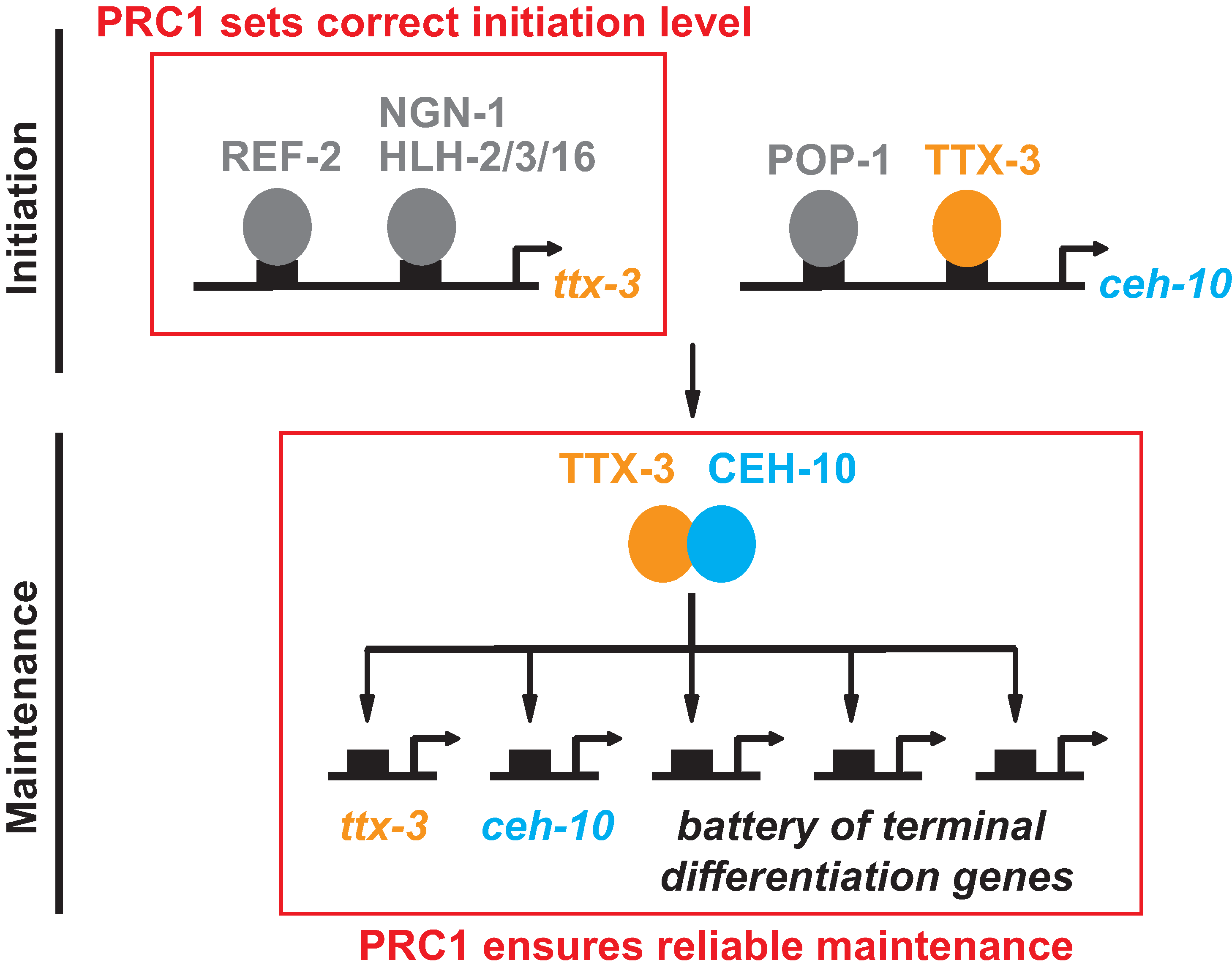

Supplement: S6 Fig — PRC1 factors help to set the correct level of ttx-3 expression during the initiation phase, and ensure the consistency of ttx-3 and ceh-10 expression during the maintenance phase. (TIF) [file pgen.1010209.s006.tif]
